# A novel cecropin B-derived peptide with antibacterial and potential anti-inflammatory properties

**DOI:** 10.7717/peerj.5369

**Published:** 2018-07-25

**Authors:** Jiarong Wang, Kun Ma, Maosen Ruan, Yujuan Wang, Yan Li, Yu V. Fu, Yonghong Song, Hongbin Sun, Junfeng Wang

**Affiliations:** 1 High Magnetic Field Laboratory, Hefei Institutes of Physical Science, Chinese Academy of Sciences, Hefei, China; 2 Key Laboratory of High Magnetic Field and Ion Beam Physical Biology, Chinese Academy of Sciences, Hefei, China; 3 The First Affiliated Hospital of Xinxiang Medical University, Xinxiang, China; 4 State Key Laboratory of Microbial Resources, Institution of Microbiology, Chinese Academy of Sciences, Beijing, China; 5 School of Chemistry and Chemical Engineering, Hefei University of Technology, Hefei, China; 6 School of Food and Biological Engineering, Zhengzhou University of Light Industry, Zhengzhou, China; 7 Institute of Physical Science and information Technology, Anhui University, Hefei, China

**Keywords:** Cecropin DH, Antibacterial activity, LPS small micelle formation, Inhibition of pro-inflammatory cytokines

## Abstract

Cecropins, originally found in insects, are a group of cationic antimicrobial peptides. Most cecropins have an amphipathic N-terminal segment and a largely hydrophobic C-terminal segment, and normally form a helix-hinge-helix structure. In this study, we developed the novel 32-residue cecropin-like peptide cecropin DH by deleting the hinge region (Alanine-Glycine-Proline) of cecropin B isolated from Chinese oak silk moth, *Antheraea pernyi*. Cecropin DH possesses effective antibacterial activity, particularly against Gram-negative bacteria, with very low cytotoxicity against mammalian cells. Interactions between cecropin DH and the highly anionic lipopolysaccharide (LPS) component of the Gram-negative bacterial outer membrane indicate that it is capable of dissociating LPS micelles and disrupting LPS aggregates into smaller assemblies, which may play a vital role in its antimicrobial activity. Using LPS-stimulated mouse macrophage RAW264.7 cells, we found that cecropin DH exerted higher potential anti-inflammatory activity than cecropin B, as demonstrated by the inhibition of pro-inflammatory cytokines nitric oxide production and secretion of tumor necrosis factor-α. In conclusion, cecropin DH has potential as a therapeutic agent for both antibacterial and anti-inflammatory applications.

## Introduction

In recent decades, antibiotic resistance has emerged as a major threat to global healthcare and food security (Furuya & Lowy, 2006), and new antibiotics are urgently needed. Antimicrobial peptides (AMPs), against which it is difficult for bacteria to develop drug resistance, have emerged as attractive candidates for treating microbial infections (Marr, Gooderham & Hancock, 2006). AMPs are important immune effectors that are present in a wide variety of organisms including mammals, insects, vertebrates, amphibians, bacteria and plants (Bulet, Stocklin & Menin, 2004; Mishra et al., 2017; Tossi, Sandri & Giangaspero, 2000; Zasloff, 2002). These short 12–100 amino acid residue peptides often contain numerous positively charged arginines and lysines. Many AMPs have an amphipathic structure that enables them to preferentially interact with anionic bacterial membranes (Hancock & Lehrer, 1998; Jenssen, Hamill & Hancock, 2006). AMPs can be classified based on secondary structure into α-helical peptides, β-sheet peptides, mixed α/β peptides and random coil peptides (Lakshmaiah Narayana & Chen, 2015). These peptides kill bacteria by disrupting cell membranes and/or interacting with internal targets (Brogden, 2005; Hancock & Sahl, 2006).

For Gram-negative bacteria, lipopolysaccharide (LPS) is the major barrier protecting against host defense molecules such as AMPs. LPS, also known as endotoxin, forms a highly anionic layer at the outer leaflet of the outer surface membrane of Gram-negative bacteria, contributing greatly to structural integrity and preventing entry of host molecules (Nikaido, 2003). LPS can be divided into three regions: a hydrophobic conserved lipid A, a highly variable hydrophilic polysaccharide known as O antigen and a core oligosaccharide that covalently links lipid A and O antigen (Raetz & Whitfield, 2002). The structure of LPS is highly conserved among Gram-negative bacteria, and it is essential for outer membrane stability (and consequently cell viability). Therefore, LPS is clearly an attractive potential target for the development of novel antimicrobial compounds. LPS also triggers the release of cytokines, and stimulates innate immune defenses, but overstimulation of innate systems may lead to fatal septic shock (Cohen, 2002). It is reported that some AMPs are able to interact with LPS with high affinity (Ding et al., 2003; Nell et al., 2006; Rosenfeld, Papo & Shai, 2006), or block the binding of LPS to LPS-binding proteins (Nagaoka et al., 2001; Nell et al., 2006).

In insects, cecropins constitute a large family of cationic AMPs that are active against Gram-positive and Gram-negative bacteria, as well as fungi, with low toxicity toward eukaryotic cells such as erythrocytes (Sato & Feix, 2006; Vizioli et al., 2000). Most cecropin-like peptides are 31–39 amino acids in length and are devoid of cysteines. They share over 50% sequence homology, and consist of an amphipathic basic N-terminal α-helical segment linked to a hydrophobic C-terminal α-helical segment by a hinge region (Boman, 1991; Lee et al., 2015). The cationic amphipathic characteristics of cecropins may contribute to their initial electrostatic contacts with polyanionic areas of the cell wall or membrane of the target microorganism (Durell, Raghunathan & Guy, 1992; Onate-Garzon et al., 2017).

In the present study, we developed a novel hinge-deletion derivative (cecropin DH) from cecropin B (KWKIFKKIEKVGRNIRNGIIKAGPAVAVLGEAKAL) that was isolated from Chinese oak silk moth, *Antheraea pernyi* (Qu et al., 1982). The antimicrobial and potential anti-inflammatory activities of cecropin DH and its interaction with LPS were characterized by a variety of biological and biophysical assays. Our results showed that the cecropin DH peptides interact with LPS lipid, and disrupt LPS aggregates into smaller assemblies. The peptides also exhibit inhibitory effect on pro-inflammatory cytokines nitric oxide (NO) production and tumor necrosis factor (TNF)-α release in LPS-stimulated mouse macrophage RAW264.7 cells. Indeed, these activities were more pronounced than those of the parent peptide cecropin B. These results strongly suggest that cecropin DH is a potent peptide antibiotic possessing potential anti-inflammatory activities.

## Materials and Methods

### Peptide preparation

Cecropin DH and its parent peptide cecropin B were synthesized by GL Biochem Ltd. (Shanghai, China) using the solid-phase synthesis technique. And the success of peptide synthesis was identified via electrospray ionization mass spectrometry. The purity of the synthetic peptides used to evaluate biological activity was higher than 95% confirmed with analytical reverse-phase high-performance liquid chromatography.

### Antimicrobial measurements and kinetics of bacteria killing

The minimum inhibitory concentrations (MICs) of cecropin DH against *Escherichia coli* ATCC 25922, *E. coli* DH5α, *Pseudomonas aeruginosa* ATCC 27853, *Bacillus subtilis* ATCC 6633, *Staphylococcus aureus* ATCC 25923 and *Micrococcus luteus* NCIMB 8166 were determined using the modified classical microtiter broth dilution method (available from http://cmdr.ubc.ca/bobh/method/modified-mic-method-for-cationic-antimicrobial-peptides/) and compared with those of cecropin B and melittin purchased from (MedChemExpress, Monmouth Junction, NJ, USA). Briefly, bacteria were incubated in Mueller Hinton Broth (MHB) medium overnight at 37 °C (30 °C for *B. subtilis*) with shaking and diluted into fresh MHB at a cell density of 5 × 10^5^ colony-forming units (CFUs)/mL. A 100 μL volume of cell suspension was dispensed in each well of a sterile 96-well plate from column 1 to column 11, and 100 μL of MHB was dispensed in column 12 for sterility control and to blank the plate scanner. A 11 μL sample of each of the peptides (10× stocks) was dissolved separately in 0.01% acetic acid and 0.2% bovine serum albumin, and added to each well (columns 1–10) to a final concentration of 100, 50, 25, 12.5, 6.25, 3.125, 1.56, 0.78, 0.39 and 0.195 μM, respectively. Column 11 without peptides served as positive controls (untreated bacteria). The plate was incubated at 37 °C (30 °C for *B. subtilis*) for 18–24 h in an incubator, and the absorbance was recorded at 590 nm. Three replicates were generated for each test sample. In the present study, the MIC was taken to be the lowest concentration of peptides that prevented visible turbidity. Then 10 μl 10^−6^ dilution of the first three wells that showed no visible growth of above overnight cultures, were plated onto Mueller Hinton Agar (MHA) plates and incubated at 37 °C (30 °C for *B. subtilis*) for 18 h. The lowest concentration of peptides that prevent any residual colony formation is the minimal bactericidal concentration.

Next, the salt sensitivity tests for cecropin DH were performed at fixed concentrations of NaCl, CaCl_2_ or MgCl_2_. MIC values against *E. coli* ATCC 25922 were measured as described above in the presence of 100 mM NaCl, 2 mM CaCl_2_ or 1 mM MgCl_2_.

The in vitro bacteria killing kinetics of cecropin DH against *E. coli* ATCC 25922 were also determined. Exponentially growing *E. coli* cells were resuspended in MHB to a density of 4 × 10^6^ CFUs/mL. Bacterial suspensions were treated at 1× and 4× MIC with peptide. At various time periods (0, 2, 3 and 6 h), 50 μL samples were removed and diluted appropriately in MHB, and CFUs were determined by spotting 100 μL of serially diluted samples onto MHB agar plates and incubating for 18 h at 37 °C.

### Measurement of hemolytic activity

The hemolytic activities of cecropin DH and cecropin B peptides were determined as the amount of hemoglobin released by the lysis of mouse red blood cells (mRBCs) (Kim et al., 2011). Fresh mRBCs were collected and centrifuged at 1,000 g for 10 min. The erythrocytes were then washed three times and resuspended in phosphate-buffered saline (PBS, pH 7.4) at 8% (v/v). Each 50 μL sample of mRBCs was incubated with 50 μL serial dilutions of peptides dissolved in PBS for 1 h at 37 °C. Samples were then centrifuged at 1,000 g for 5 min at 4 °C, and the supernatant was transferred to a 96-well microtiter plate. The release of hemoglobin was monitored by measuring the absorbance of the supernatant at 405 nm. No hemolysis (blank) and 100% hemolysis controls consisting of mRBCs in PBS and 0.1% (v/v) Triton X-100, respectively, were included. Percentage hemolysis was calculated as follows:

$${\rm{Hemolysis}}\left( {\rm{\% }} \right) = \left[ {{{\left( {{\rm{O}}{{\rm{D}}_{{\rm{405\ nm}}}}{\rm{sample}} - {\rm{O}}{{\rm{D}}_{{\rm{405\ nm}}}}{\rm{zero\ lysis}}} \right)} \over {\left( {{\rm{O}}{{\rm{D}}_{{\rm{405\ nm}}}}{\rm{100\%\ lysis}} - {\rm{O}}{{\rm{D}}_{{\rm{405\ nm}}}}{\rm{zero\ lysis}}} \right)}}} \right]{\rm{ \times 100}}{\rm{.}}$$

### Cytotoxicity assay

The cytotoxicity of cecropin DH and cecropin B peptides against RAW264.7 mouse macrophage cells (from the Cell Resource Center of the Shanghai Academy of Life Sciences, Chinese Academy of Sciences) was determined using a CCK-8 cell proliferation and cytotoxicity assay kit (BestBio, Shanghai, China). RAW264.7 cells were cultured in Dulbecco’s modified Eagle’s medium (DMEM; Corning Cellgro, Manassas, Virginia, USA) supplemented with antibiotics (100 U/mL penicillin and 100 μg/mL streptomycin; Sangon Biotech, Shanghai, China) and 10% fetal bovine serum (Gibco, Grand Island, NY, USA) at 37 °C in a humidified chamber under a 5% CO_2_ atmosphere. Cells were pre-seeded on a 96-well plate at a density of ∼6 × 10^4^ to 1 × 10^5^ cells/mL and cultured overnight. Increasing concentrations of the peptides (0.195–100 μM) in DMEM medium were added, and wells containing cells without peptides served as controls. After incubating plates for 1 day, 10 μL of CCK-8 solution was added to each well and plates were incubated for an additional 2.5 h at 37 °C. The absorbance at 450 nm was measured using a microplate autoreader (SpectraMax i3x; Molecular Devices, San Jose, CA, USA).

### BODIPY-TR-cadaverine displacement assay

The binding affinity of cecropin DH to the lipid A portion of bacterial LPS from *E. coli* 055:B5 purchased from (Sigma-Aldrich, St. Louis, MO, USA) was determined using the fluorescent dye BODIPY-TR-cadaverine (BC; from ThermoFisher Scientific Inc., Waltham, MA, USA) displacement assay (Ma et al., 2015; Sautrey et al., 2014), in which the fluorescence of the probe is self-quenched upon binding to cell-free LPS, while fluorescence is emitted when LPS binds to the peptides. Briefly, 100 μL solutions in 20 mM TRIS and 100 mM NaCl (pH 7.4) containing equal volumes of LPS-probe mixture (final concentrations of 25 μg/mL for LPS and 2.5 μg/mL for BC) and 1.56–50 μM peptide were incubated in 96-microwell black plates in the dark for 0.5 h. Fluorescence was measured using a microplate autoreader with excitation at 580 nm and emission at 620 nm. Values were converted to %ΔF (AU) using the following equation:

$${\rm{\% \Delta F }}\left( {{\rm{AU}}} \right)=\left[ {{{\left( {{{\rm{F}}_{{\rm{obs}}}} - {{\rm{F}}_{\rm{0}}}} \right)} \over {\left( {{{\rm{F}}_{{\rm{100}}}} - {{\rm{F}}_{\rm{0}}}} \right)}}} \right]{\rm{ \times 100,}}$$

where F_obs_ is the observed fluorescence of BC with LPS-peptide interaction, F_0_ is the fluorescence of BC with LPS in the absence of peptide (corresponding to no displacement of the probe) and F_100_ is the fluorescence upon addition of 10 μg/mL polymyxin B purchased from (Sigma-Aldrich, St. Louis, MO, USA) to the LPS-probe mixture (corresponding to maximal displacement of the probe).

### Disassociation of LPS

Interactions between cecropin DH and fluorescein isothiocyanate (FITC)-conjugated LPS from *E. coli* 055:B5 purchased from (Sigma-Aldrich, St. Louis, MO, USA) were analyzed by exciting FITC-LPS (0.5 μM) at 480 nm in 20 mM TRIS and 100 mM NaCl (pH 7.4) in the absence and presence of different concentrations of peptide (0.78, 1.56, 3.125, 6.25, 12.5, 25, 50 and 100 μM). Fluorescence emission spectra at 515 nm were recorded using a SpectraMax i3x microplate autoreader (Molecular Devices, San Jose, CA, USA).

### Static light scattering assay

To obtain information on the particle size distribution of LPS micelles in the absence and presence of cecropin DH, static light scattering (SLS) measurements were performed on a Malvern Zetasizer μV instrument (Malvern, UK). Measurements were made for LPS micelles at a final concentration of 1 mg/mL mixed with 0.25 mM peptides. LPS and peptide were dissolved in TRIS buffer (20 mM TRIS, 100 mM NaCl, pH 7.4), and all solutions were filtered and degassed prior to use. Data were acquired at 298 K using disposable cuvettes. Scattering data were collected at 90° and analyzed using the software supplied with the instrument.

### Examination of LPS micelle damage by transmission electron microscopy

Transmission electron microscopy (TEM) was performed to observe the influence of peptides on the morphology of LPS micelles. A total of 50 μL solutions containing 0.25 mM cecropin DH and 1 mg/mL LPS micelles in Milli-Q water were incubated at room temperature overnight. Untreated LPS micelles were used as controls. After incubation, a drop of the mixture was deposited onto a carbon-coated grid, negatively stained with phosphotungstic acid and visualized using a Tecnai G2 Spirit BioTWIN 120 kV TEM instrument (FEI, Hillsboro, Oregon, USA).

### Circular dichroism spectroscopy

Circular dichroism (CD) experiments were performed at room temperature with a Chirascan qCD spectropolarimeter (Applied Photophysics Ltd., Surrey, UK). Spectra were recorded at wavelengths ranging from 190 to 240 nm. CD spectroscopy was used to investigate the secondary structure of cecropin DH in the absence and presence of 2 mg/mL LPS and *E. coli* ATCC 25922 bacterial cells in 10 mM sodium phosphate buffer (pH 7.4). The peptide concentration was 0.2 mg/mL. The CD spectra of cecropin DH in sodium phosphate buffer, in LPS or *E. coli* cells environment were obtained by subtracting the peptide control spectrum. Spectra were collected in quartz cuvettes with a 0.5 mm path length and averaged across three consecutive scans. A 1 nm data pitch, 0.4 s time-per-point and 1 nm bandwidth were generally used. In addition, *E. coli* bacterial cell cultures for CD experiments were grown in MHB medium at 37 °C overnight, harvested (4,000 g, 8 min) and resuspended in 10 mM sodium phosphate buffer (pH 7.4) at OD_600_ values of 4.0, 2.0, 1.0 and 0.5. Peptide solution was added to the resuspended *E. coli* cells to give a final OD_600_ of 2.0, 1.0, 0.5 and 0.25, and samples were incubated at room temperature for 4 h (Avitabile, D’Andrea & Romanelli, 2014).

### Nuclear magnetic resonance experiments

For saturation transfer difference (STD) experiments, the nuclear magnetic resonance (NMR) sample contained 1 mM cecropin DH, 0.3 mg/mL LPS, in 10 mM sodium phosphate D_2_O buffer at pH 5.8. STD experiments were recorded on a Bruker 500 MHz spectrometer at a temperature of 298 K with 1,024 scans. Selective saturation of LPS resonances was performed by selective irradiation at frequency at −2.0 ppm (on-resonance) or 40 ppm for the reference spectrum (off-resonance). A cascade of 44 selective Gaussian-shaped pulses was applied for a total saturation time of 2 s. The low power Gaussian pulses had a duration of 45 ms and inter-pulse delays of 450 μs. The difference spectrum, which contains signals arising from the saturation transfer, was obtained by subtracting the off-resonance spectrum from the on-resonance spectrum by phase cycling.

^31^P nuclear magnetic resonance spectra of LPS were recorded on a Bruker 850 MHz spectrometer at 298 K. Interactions of peptides with LPS were examined by recording a series of one-dimensional ^31^P NMR spectra of LPS, whereby 4 mg/mL LPS in 20 mM TRIS (pH 7.4) and 100 mM NaCl was titrated with various peptide concentrations (0.2, 0.4 and 0.6 mM).

### Reverse-transcription PCR assay

RAW264.7 murine macrophage cells were plated in six-well plates (5 × 10^5^ cells/well) and cultured overnight. Cells were then stimulated with or without 200 ng/mL LPS (negative controls) in the presence or absence of peptides (10 μM) for 3 h. Total RNA was prepared using an RNAprep Kit (TIANGEN, Beijing, China), and equal amounts of total RNA were reverse-transcribed into cDNA using a RevertAid first-strand cDNA synthesis kit (ThermoFisher Scientific Inc., Waltham, MA, USA) according to the manufacturer’s protocol. Products were amplified from cDNAs by PCR using the following specific primers: interleukin-1β (IL-1β) (forward = 5′-CTGTCCTGATGAGAGCATCC-3′, reverse = 5′-TGTCCATTGAGGTGGAGAGC-3′); interleukin 6 (IL-6) (forward = 5′-ACAAGTCCGGAGAGGAGACT-3′, reverse = 5′-GGATGGTCTTGGTCCTTAGC-3′); macrophage inflammatory protein (MIP)-1 (forward = 5′-ATGAAGCTCTGCGTGTCTGC-3′, reverse = 5′-TGAGGAGCAAGGACGCTTCT-3′); MIP-2 (forward = 5′-ACACTTCAGCCTAGCGCCAT-3′, reverse = 5′-CAGGTCAGTTAGCCTTGCCT-3′); TNF-α (forward = 5′-GTTCTGTCCCTTTCACTCACTG-3′, reverse = 5′-GGTAGAGAATGGATGAACACC-3′); inducible nitric-oxide synthase (iNOS) (forward = 5′-CTGCAGCACTTGGATCAGGAACCTG-3′, reverse = 5′-GGGAGTAGCCTGTGTGCACCTGGAA-3′); glyceraldehyde-3-phosphate, used as an internal control (forward = 5′-ACCACAGTCCATGCCATCAC-3′, reverse = 5′-TCCACCACCCTGTTGCTGTA-3′). Thermal cycling consisted of an initial denaturation step of 5 min at 94 °C, followed by 35 cycles of denaturation at 94 °C for 1 min, annealing at 55 °C for 1.5 min and extension at 72 °C for 1 min, and a final extension step of 10 min at 72 °C.

### Measuring nitrite production in LPS-stimulated RAW264.7 cells

RAW264.7 murine macrophage cells (1.5 × 10^5^) cultured in DMEM containing 10% fetal bovine serum, 100 U/mL penicillin and 100 μg/mL streptomycin were plated and adhered to a 96-well culture plate. After stimulating with LPS (200 ng/mL) in the presence or absence of peptides (10 μM) for 24 h, culture medium was collected to determine nitrite levels using Griess reagent (Beyotime, Jiangsu, China) according to the manufacturer’s instructions. The absorbance at 540 nm was measured using a SpectraMax i3x microplate reader (Molecular Devices, San Jose, CA, USA) and converted to nitrite concentration by referring to a standard curve generated using NaNO_2_.

### Quantification of pro-inflammatory cytokine production by LPS-stimulated RAW264.7 cells

RAW264.7 murine macrophage cells were seeded in 96-well plates (5 × 10^4^ cells/well) and incubated overnight. Cells were pretreated with peptides (10 μM) and incubated at 37 °C for 1 h before the addition of 20 ng/mL LPS. After incubation for another 6 h, supernatants were harvested for TNF-α and IL-6 analyses using commercially available mouse TNF-α and IL-6 enzyme-linked immunosorbent assay (ELISA) kits (DAKEWE, Shanghai, China) according to the manufacturer’s protocols. The absorbance at 450 nm was measured using a SpectraMax i3x microplate reader (Molecular Devices, San Jose, CA, USA).

### Statistical analysis

The statistical significance of differences between samples were analyzed by one-way analysis of variance, followed by Bonferroni’s multiple comparison test (GraphPad Prism; GraphPad Software, San Diego, CA, USA). Differences with *p* < 0.05 were considered statistically significant.

## Results

### Antimicrobial activity

The antimicrobial activities of cecropin DH were examined against three representative Gram-negative (*E. coli* ATCC25922, *E. coli* DH5α and *P. aeruginosa*) and three Gram-positive (*B. subtilis*, *S. aureus* and *M. luteus*) bacterial strains (Table 1), and compared with the activities of melittin, which is known to have profound antibacterial activities. The antimicrobial activities of the parent peptide cecropin B are also listed for comparison. Overall, as indicated in Table 1, cecropin DH peptides displayed a good inhibitory effect on *E. coli*, *P. aeruginosa*, *B. subtilis* and *M. luteus*, which was comparable to those of cecropin B and melittin. Interestingly, both cecropins in this study exhibited almost no activity against *S. aureus*, with MICs >100 μM.

**Table 1:** Minimal inhibitory concentrations and minimal bactericidal concentration of peptides against standard bacterial strains.

Gram-negative				Gram-positive			
	MIC (MBC)^a^ (μM)				MIC (MBC) (μM)		
Bacterial strains	Cecropin DH	Cecropin B	Melittin	Bacterial strains	Cecropin DH	Cecropin B	Melittin
*E. coli* ATCC25922	3.13 (6.25)	1.56 (1.56)	6.25 (6.25)	*B. subtilis*	3.13 (3.13)	6.25 (6.25)	1.56 (1.56)
*E. coli* DH5α	1.56 (3.13)	0.78 (0.78)	3.13 (6.25)	*S. aureus*	>100 (>100)	>100 (>100)	3.13 (3.13)
*P. aeruginosa*	6.25 (12.5)	3.13 (12.5)	6.25 (25)	*M. luteus*	1.56 (1.56)	0.78 (0.78)	3.13 (3.13)
GM^b^	3.65	1.82	5.21	GM	68.23	69.01	2.61
MHC^c^	400	400	0.78	MHC	400	400	0.78
Therapeutic index^d^ (MHC/GM)	109.59	219.78	0.15	Therapeutic index^d^ (MHC/GM)	5.86	5.80	0.30

**Notes:**

a MICs was determined as the lowest concentration of peptides that prevented visible turbidity. MBC was taken to be the lowest concentration of peptides that prevent any residual colony formation. When no antimicrobial activity was observed at 100 μM, a value of 200 μM was used to calculate the therapeutic index.

b The geometric mean (GM) of the MIC was obtained from bacterial strains are shown.

c The MHC is the minimum hemolytic concentration that caused 10% hemolysis of mouse red blood cells (mRBCs). The MHC value of melittin is from reference (Kim et al., 2011). When it did not reach 10% hemolysis at 200 μM, a value of 400 μM was used to calculate the therapeutic index.

d Therapeutic index is the ratio of the MHC value over GM.

**Figure 1:**
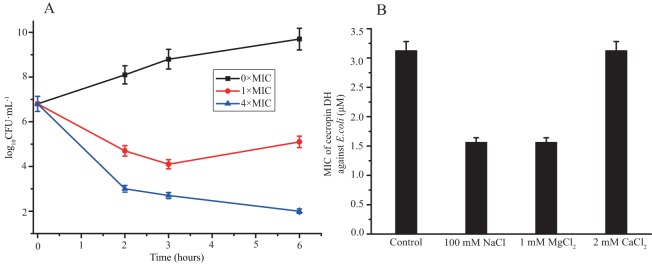
Antibacterial activity of cecropin DH. (A) Loss of viability of *Escherichia coli* ATCC25922 after incubation with cecropin DH. Bacteria were incubated for different incubation times at concentrations of 1× and 4×MIC. (B) Effect of different salts on MIC values of cecropin DH against *E. coli* ATCC25922.

The bacteria killing activity of cecropin DH against *E. coli* was also monitored by assessing cell viability loss after incubation with peptide at 1× and 4× MIC for 6 h. As shown in Fig. 1A, cecropin DH exerted an obvious dose-dependent antibacterial activity within 6 h. After 2 h of incubation, a reduction in the number of CFUs was approximately 2 log with peptide concentration of 1×MIC, and a rapid loss of cell viability (3.8 log reduction in the number of CFUs) was observed at 4×MIC. The number of CFUs recovered for the *E. coli* incubated with 1×MIC peptide after 6 h incubation. While cecropin DH at 4×MIC preserves its antimicrobial activity at hour 6. The salt sensitivity of the antimicrobial activity was estimated by monitoring the MICs of peptides in the presence of 100 mM NaCl, 1 mM MgCl_2_ and 2 mM CaCl_2_, which were chosen for their biological relevance. For example, 100 mM NaCl was reported to present in the airway surface fluid of healthy subjects and patients with cystic fibrosis (Scudiero et al., 2010). As shown in Fig. 1B, the MIC values of cecropin DH were not significantly affected by the presence of different salts. The antibacterial activity against *E. coli* was largely unchanged with 2 mM CaCl_2_, and even doubled in the presence of 100 mM NaCl and 1 mM MgCl_2_.

### Hemolysis and cytotoxicity

**Figure 2:**
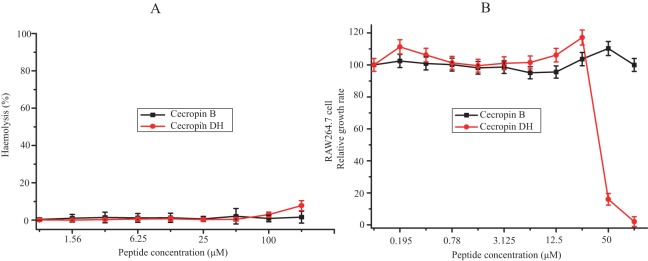
Cytotoxic effects against mammalian cells. (A) Dose-response curve for the hemolytic activity of peptides toward mouse erythrocytes. (B) Cytotoxicity of peptides toward macrophage-derived RAW264.7 cells.

The hemolysis activity of cecropin DH and cecropin B against mRBCs was assessed as a measurement of peptide toxicity to eukaryotic cells. Hemolysis percentage was quantified at a concentration range from 0.78 to 200 μM. As shown in Fig. 2A, although a dose-dependent hemolytic activity was observed, in general cecropin DH induced very little hemolytic activity. At concentrations of 100 or 200 μM, cecropin DH showed 2.9% and 7.8% red cell hemolysis, respectively. At a concentration below 100 μM, almost no hemolytic activity was observed. Similarly, cecropin B lacked hemolytic activity, even at concentration as high as 200 μM. As a comparison, melittin caused 10% hemolysis at concentration as low as 0.78 μM (Kim et al., 2011; Pandey et al., 2010). The cytotoxicity of these two peptides was evaluated using RAW264.7 murine macrophage cells with various peptide concentrations (0.195–100 μM), and the effects on cell growth were subsequently evaluated using CCK-8 cell viability assay reagent. As illustrated in Fig. 2B, neither of the peptides affected the viability of RAW264.7 cells at concentrations less than 25 μM, and the survival rates of RAW264.7 cells were greater than 95%. Cecropin DH showed high cytotoxicity at concentrations above 25 μM, and an analysis of dose-response curves by GraphPad Prism 7.00 software yield an IC_50_ of 46.34 μM, much higher than its MIC value (Table 1).

### Disruptions of LPS aggregates

**Figure 3:**
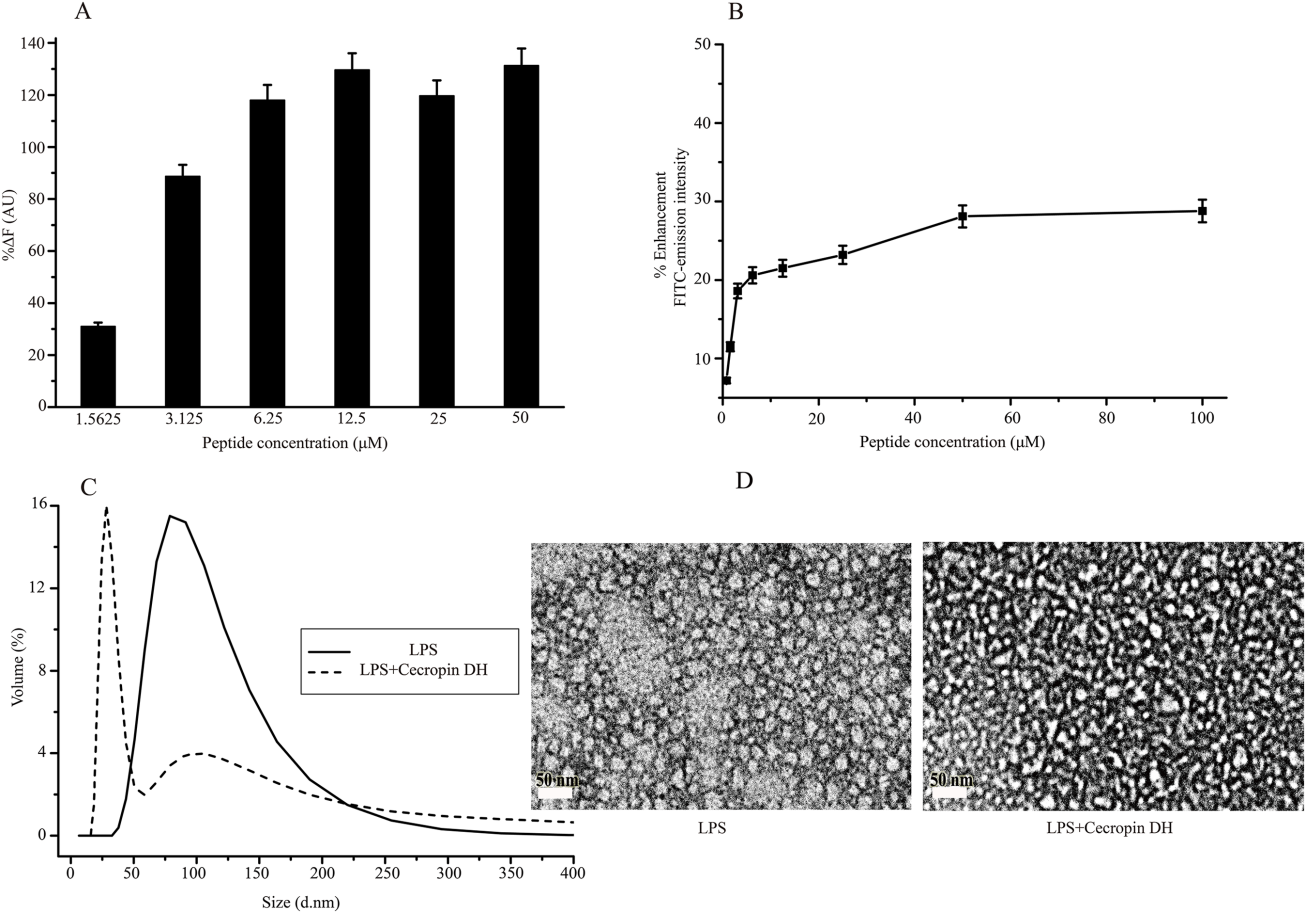
Structural disruption of lipopolysaccharide (LPS) micelles by cecropin DH. (A) Binding affinity of cecropin DH to LPS based on displacement assays with BODIPY-TR-cadaverine (BC) fluorescent dye. (B) Enhancement of the intensity of 0.5 μM fluorescein isothiocyanate (FITC)-labelled LPS with increasing concentrations of cecropin DH (0.78, 1.56, 3.125, 6.25, 12.5, 25, 50 and 100 μM). AU, absorbance unit. (C) Size distribution of LPS micelles (1 mg/mL) in the absence and presence of 0.25 mM cecropin DH by static light scattering (SLS) measurements. (D) Transmission electron microscopy (TEM) analysis of negatively stained LPS micelles (1 mg/mL) with and without treatment with 0.25 mM cecropin DH.

Lipopolysaccharide forms super-molecular structures in aqueous solution, which provides a convenient in vitro model for studying the effects of cecropin DH. First, the binding ability of cecropin DH to LPS was investigated using the BC fluorescence-based displacement assay. As shown in Fig. 3A, cecropin DH induced a significant dose-dependent BC fluorescence displacement. The BC displacement percentage was 30% at a low peptide concentration of 1.56 μM, and increased to 89% at 3.125 μM. At a cecropin DH concentration above 6.25 μM, fluorescence recovered to 100%, reflecting potent binding to the lipid A region of LPS. For FITC-conjugated LPS, the fluorescence intensity of FITC was largely quenched due to the formation of LPS soluble aggregates in solution (Tobias et al., 1995). As shown in Fig. 3B, the fluorescence intensity of FITC-LPS changed with increasing peptide concentration. Again, the addition of active peptide increased the fluorescence intensity in a concentration-dependent manner, indicating possible LPS dissociation and/or structural disorganization of LPS micelles. Similar phenomena have been observed with other AMPs in which binding of proteins or peptides alleviates quenching and enhances FITC fluorescence (Bhunia et al., 2009a; Kim et al., 2011).

The effect of cecropin DH on LPS aggregates can be conveniently characterized by SLS. As shown in Fig. 3C, LPS in aqueous solution assembles into large aggregates with a broad size distribution from 50 to 200 nm. After the addition of peptides, the population of large LPS micelles decreased, and a new component of ∼30 nm with a much narrower size distribution appeared. These smaller LPS micelles were further verified by TEM imaging (Fig. 3D). These observations demonstrated that the AMP cecropin DH destabilizes LPS micelles, causing them to break into smaller assemblies.

### Interactions between cecropin DH and LPS

**Figure 4:**
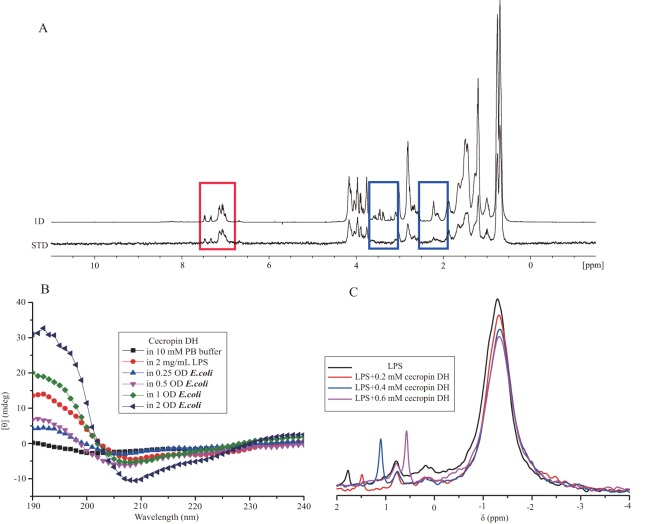
Interaction of cecropin DH with LPS. (A) Superposition of the saturation transfer difference (STD) NMR spectrum and the reference NMR spectrum of 1 mM cecropin DH in 0.3 mg/mL LPS (in 10 mM sodium phosphate D_2_O buffer, pH 5.8) at 298 K. The red box shows the STD effect of the aromatic ring protons of Trp2 and Phe5, and the blue box shows areas with no STD effect. (B) Secondary structures of 0.2 mg/mL cecropin DH in the absence and presence of LPS and *E. coli* bacteria cells in 10 mM sodium phosphate buffer (pH 7.4) at 298 K. (C) One-dimensional ^31^P NMR spectra of 4 mg/mL LPS and spectra after addition of different concentrations of cecropin DH (pH 7.4) at 298 K.

Further experiments were performed to investigate the interactions of cecropin DH and LPS. STD-NMR spectra were recorded on 1 mM peptide samples with 0.3 mg/mL LPS (in 10 mM sodium phosphate D_2_O buffer at pH 5.8). In the STD-NMR experiments, selective saturation pulses were applied at LPS resonances, and magnetic saturation was subsequently transferred to LPS-bound peptides. Saturation difference spectra were generated from the intensity difference between LPS-bound peptide and free peptide signals. Figure 4A shows the ^1^H spectrum of free peptides and the STD spectrum of peptides bound to LPS. A relatively strong STD effect of cecropin DH was observed for the aromatic ring protons of Trp2 and Phe5 (6–8 ppm), as well as a number of aliphatic side chain proton resonances (0–5 ppm) (Bhunia, Ramamoorthy & Bhattacharjya, 2009b; Kim et al., 2011; Lee et al., 2015), implying that these hydrophobic side chains are closely associated with LPS micelles.

Direct evidence for the formation of α-helical structure in cecropin DH was provided by CD spectroscopy. CD spectra of cecropin DH dissolved in phosphate buffer (Fig. 4B) or water (Fig. S1) exhibit a negative peaks at 198 nm, indicating disordered conformations in aqueous buffer. By contrast, in 2 mg/mL LPS solution (Fig. 4B), and in the presence of other hydrophobic reagents such as trifluoroethanol, dodecylphosphatidylcholine and sodium dodecyl sulfate (SDS; Fig. S1), two negative minimum peaks at 208 and 222 nm appear, indicating that cecropin DH adopts an α-helical conformation in hydrophobic environments. When cecropin DH peptides were incubated with *E. coli* cells, α-helical structure also formed, and the α-helical content was dependent on cell concentration. This is due to the fact that more LPS molecules are available for peptide binding with the increment of cell concentration. Fourier-transform infrared (FTIR) spectra subsequently provided additional evidence; in the presence of LPS, the appearance of a peak at 1,655 cm^−1^ in the amide I region indicates an α-helical conformation for cecropin DH (Fig. S2A). Furthermore, a peak at ∼1,675 cm^−1^ was observed, which is characteristic of carbonyls not involved in hydrogen-bonded structures (Sun et al., 2015).

^31^P nuclear magnetic resonance signals from phosphate groups which locate within the lipid A moiety and inner sugar core region, provide a convenient probe of the local structural and environmental conditions around the LPS head group. Figure 4C shows an overlay of ^31^P NMR spectra of 4 mg/mL LPS in the absence and presence of cecropin DH peptide at different concentrations (0.2, 0.4 and 0.6 mM). The ^31^P NMR spectra of LPS micelles exhibit a strong peak close to −1.30 ppm and two weak peaks at approximately 0.8 and 1.78 ppm, corresponding to the diphosphate and monophosphate groups of LPS, respectively (Bera et al., 2015; Strain, Fesik & Armitage, 1983). As the molar ratios of peptides to LPS increased, the most significant and obvious change happens at resonance of 1.78 ppm, which became sharper and upfield-shifted to 1.48, 1.06 and 0.52 ppm respectively. This suggests that the LPS monophosphate group may be directly involved in peptide binding and undergo dynamics at fast exchange regime. The changes in strong peak at −1.30 ppm and peak at 0.8 ppm were relatively minor. The observed line broadening of ^31^P resonances is caused by the chemical or conformational exchange processes between different states of LPS-peptide complexes.

### Inhibition effect of peptides on LPS-induced pro-inflammatory cytokine production

**Figure 5:**
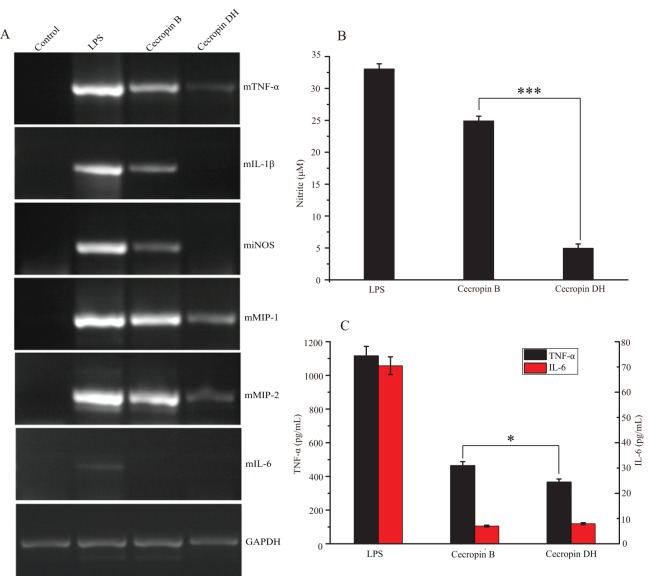
Effect of peptides on LPS-stimulated pro-inflammatory mediators in RAW264.7 cells. (A) Effects of cecropin B and cecropin DH on mRNA levels of inflammatory cytokines in 200 ng/mL LPS-stimulated RAW264.7 cells. Total RNA was analyzed for the expression of TNF-α, IL-1β, iNOS, MIP-1, MIP-2, IL-6 and GAPDH (loading control) by RT-PCR. (B) Effects of cecropin B and cecropin DH on NO production in 200 ng/mL LPS-stimulated RAW264.7 cells. (C) Effects of cecropin B and cecropin DH on TNF-α and IL-6 release from RAW264.7 cells stimulated with 20 ng/mL LPS. *p < 0.05, ***p < 0.001.

Lipopolysaccharide, also termed endotoxin, is the major component of the outer membrane of Gram-negative bacteria, and its release can cause strong immunogenicity and provoke immune responses in immunocytes. LPS can induce the expression of iNOS and the pro-inflammatory cytokines TNF-α, IL-1β, MIP-1, MIP-2 and IL-6 in macrophages. Therefore, we investigated the effects of cecropin DH on the expression of iNOS and pro-inflammatory cytokines induced in RAW264.7 macrophages by stimulating with 200 ng/mL LPS over 3 h by Reverse-transcription PCR. As shown in Fig. 5A, cecropin DH was more effective than cecropin B for suppressing iNOS expression, and was superior for suppressing all pro-inflammatory cytokine genes except IL-6.

We then evaluated LPS-stimulated NO production and cytokine production in RAW264.7 macrophages. RAW264.7 cells were stimulated with 200 ng/mL LPS in the presence of the 10 μM peptide. NO production was determined by the Griess method that detects nitrite accumulation in the culture medium. As illustrated in Fig. 5B, 200 ng/mL LPS induced 33 μM nitrite release. Compared with cecropin B, the addition of cecropin DH was more effective at inhibiting NO production, which was decreased by 85%. The inhibitory effect of cecropin DH on the release of TNF-α and IL-6 in LPS (20 ng/mL)-stimulated RAW264.7 cells was also investigated using commercially available ELISA kits. A quantitative analysis of TNF-α and IL-6 concentrations revealed that cecropin DH clearly inhibited both TNF-α and IL-6 production in LPS-simulated RAW264.7 cells (Fig. 5C). A 20 ng/mL dose of LPS alone induced 1,116 pg/mL TNF-α, and 10 μM cecropin DH blocked its release by 67.2%, whereas cecropin B blocked the release by 58.4%. With respect to IL-6, LPS (20 ng/mL) alone induced 70.5 pg/mL, cecropin DH blocked its release by 88.7% and cecropin B blocked the release by 90.1%. These data are in good agreement with the observed inhibition of the expression of iNOS, TNF-α and IL-6 genes by the peptides (Fig. 5A). In general, the novel peptide cecropin DH exhibited more potent effect on reducing the inflammatory response than its parent peptide cecropin B.

## Discussion

Cecropins are a family of cationic AMPs, the first members of which were isolated from the immunized hemolymph of *Hyalophora cecropia* pupae (Steiner et al., 1981). Most cecropin-like peptides share similar amphipathic α-helix-flexible region-hydrophobic α-helix structural motifs. In the present study, we developed cecropin DH by deleting the flexible hinge sequence (Ala22-Gly23-Pro24) of the AMP cecropin B. Antimicrobial assays showed that cecropin DH exhibited potent antibacterial activity against five standard bacterial strains (*E. coli* ATCC 25922, *E. coli* DH5α, *P. aeruginosa* ATCC 27853, *B. subtilis* ATCC 6633 and *M. luteus* NCIMB 8166). It was proposed that the antibacterial activity in cecropin-like model peptides requires the flexible region between the N-terminal amphipathic α-helix and the C-terminal hydrophobic α-helix (Fink et al., 1989; Oh et al., 1999). On contrary to this mechanism, however, the cecropin-type peptide cecropin P1 was reported to form a single straight α-helix in a HFIP/water solution and display strong antibacterial activity (Sipos, Andersson & Ehrenberg, 1992). Herein, the cecropin DH lacking the hinge region of cecropin B also exhibited high antimicrobial activities against *E. coli*, *P. aeruginosa*, *B. subtilis* or *M. luteus*, with MICs comparable with those of the parent peptide cecropin B (Table 1). Consistent with previous studies, both cecropin DH and its parent peptide cecropin B exhibited almost no activity against Gram-positive bacterial *S. aureus*. Nevertheless, it is possible to convert cecropin into an antimicrobial agent against *S. aureus*. As a matter of fact, cecropin-melittin hybrids have been constructed which were active against *S. aureus* and remained nonhemolytic (Boman et al., 1989; Ebbensgaard et al., 2015; Wade et al., 1992).

The therapeutic applications of AMPs lie in their ability to effectively kill bacterial cells without exhibiting significant cytotoxicity toward mammalian cells. Our current research demonstrated that, at its MIC value, cecropin DH did not exert cytotoxic effects on mRBCs or mouse macrophage RAW264.7 cells (Fig. 2). The cytotoxic property is usually conveyed by the concept of the therapeutic index, which is the ratio of the MHC value over GM value. A high therapeutic index is an indication of two characteristics of the peptides, a high MHC (low hemolysis) and a low MIC (high antimicrobial activity). The therapeutic index of cecropin DH is 109.59 against Gram-negative bacteria and 5.86 against Gram-positive bacteria, indicating cecropin DH is primarily an antimicrobial agent against Gram-negative bacteria (Table 1). The therapeutic index of cecropin DH against Gram-negative bacteria is about twofold less than that of cecropin B (219.78), but is still much higher than melittin (0.15). It is generally believed that most cationic AMPs are salt-sensitive, with antimicrobial activity reduced or lost at increasing salt concentrations (Huang et al., 2011; Scudiero et al., 2010; Tomita et al., 2000). However, in the present work the antimicrobial capacity of cecropin DH was not affected, and was even increased in the presence of different salts (Fig. 1B). Thus, although the underlying mechanism is not clear, this class of compounds may provide a promising new lead for novel antibacterial agents.

During bacterial growth, cell death or disruption of bacterial membranes by antibiotic treatment, LPS (endotoxin) can be released from the cell wall and elicit strong innate immune responses in animals (Beutler, 2000; Heumann & Roger, 2002). Once LPS is released into the blood system, it can trigger monocytes and phagocytic cells to secrete large amounts of various pro-inflammatory cytokines such as TNF-α, IL-6, IL-1β and various others that contribute to the pathophysiology of septic shock and other immune diseases (Aderem & Ulevitch, 2000; Sun & Shang, 2015). In recent years, numerous AMPs have been described that not only show broad-spectrum antimicrobial activity, but also inhibit the release of pro-inflammatory cytokines such as cecropin A, cecropin-like peptide papiliocin, LL-37-derived peptide and cathelicidin-PY (Lee, Shin & Kim, 2015; Kim et al., 2011; Lee et al., 2015; Rajasekaran, Kim & Shin, 2017; Wei et al., 2013). In the present study, the potential anti-inflammatory activity of cecropin DH was investigated and compared with that of cecropin B. At nontoxic concentrations, cecropin DH inhibited NO production to a greater extent, and triggered more substantial inhibition on TNF-α secretion by LPS-stimulated mouse macrophage-derived RAW264.7 (Figs. 5B and 5C). As previously reported, this could be involved in Toll-like receptor pathways in which LPS stimulates TLR4 receptors, then recruit several downstream signaling molecules. The different effects of cecropin DH and cecropin B on inhibiting pro-inflammatory cytokines may be due to their varying abilities in LPS binding. The association of cecropin DH to LPS molecules interferes or blocks the interaction of LPS to LPS-binding protein, CD14, MD2 or TLR4, ultimately suppressing the downstream signaling related with TLR4 (Kim et al., 2011; Wei et al., 2013; Suzuki et al., 2011).

Lipopolysaccharide covers >70% of the outer leaflet of Gram-negative bacteria and forms a permeability barrier that must be overcome by broad-spectrum AMPs. A variety of biophysical techniques have been employed to investigate the interactions between AMPs and LPS/mimetic membranes to understand the mechanisms of membrane recognition and perturbation by AMPs (Bhunia et al., 2010; Domadia et al., 2010; Kushibiki et al., 2014). Herein, destabilization of LPS aggregates by cecropin DH was evaluated by FITC-conjugated LPS fluorescence assays, SLS and TEM (Fig. 3). Cecropin DH caused significant disaggregation of LPS micelles into smaller-sized particles. Similar morphological changes have been reported in LPS vesicle systems (Da Silva & Teschke, 2003; Papo & Shai, 2005). The micellization and decrease in vesicle size caused by AMPs, like PGLa and synthesized amphipathic peptide 4D-K_5_L_7_, may lead to a loosening of the bacterial outer membrane and the formation of transient “cracks” that allow the passage of a variety of molecules. In this study, cecropin DH exerted strong activity against Gram-negative organisms, probably due to its ability to breach the LPS-mediated barrier by interacting with LPS, and using large structural perturbations of LPS aggregates.

Many AMPs are believed to form helical structures in LPS micelles (Kim et al., 2011; Rajasekaran, Kim & Shin, 2017; Sun et al., 2015). The AMP HAL-2 adopts a β-sheet conformation and eventually forms amyloid aggregates that are essential for its antimicrobial activity (Wang et al., 2014). Herein, cecropin DH displayed a typical α-helical conformation in membrane-mimicking environments (Fig. S1) and *E. coli* bacterial cells (Fig. 4B). The change from a random coil-like conformation to a more folded structure could be important for its antimicrobial activity. STD results revealed that Trp2 and Phe5, along with a number of aliphatic side chains, produce a STD effect in the presence of LPS (Fig. 4A). Trp2 and Phe5 are highly conserved residues in cecropin-like peptides, and are reported to be important for interaction with negatively charged bacterial cell membranes, as well as LPS in infected cell membranes (Kim et al., 2011; Lee et al., 2015). Additionally, aromatic residues in designed β-boomerang AMPs appeared to serve as a lock to secure the insertion of the peptide into LPS (Bhunia et al., 2009a). As reflected in the ^31^P spectra (Fig. 4C) and FTIR spectra (Fig. S2B), LPS experiences complicated conformational and dynamic changes. Similar structural perturbations and disorder of LPS micelles have also been observed for other AMPs such as pardaxin (Bhunia et al., 2010) and indolicidin (Bera et al., 2015).

## Conclusion

Herein, we developed and characterized a novel cecropin-like peptide cecropin DH, which exhibited high antibacterial activity, and higher inhibition activity on pro-inflammatory cytokines than the parent peptide, without significant cytotoxicity toward mammalian cells. Mechanistic analysis suggested that interaction between cecropin DH and LPS disrupts the bacterial outer membrane by forming small cecropin DH-LPS assemblies. Although more detailed mechanistic studies are needed, the observed fine balance between antibacterial activities and reduction in the inflammatory response of cecropin DH could assist the design of novel AMPs for future therapeutic purposes.

## Supplemental Information

10.7717/peerj.5369/supp-1 Supplemental Information 1 Fig. S1. Circular dichroism (CD) spectra of cecropin DH. CD spectra of cecropin DH (0.2 mg/mL) in H_2_O, 100 mM sodium dodecyl sulphate (SDS), 30% trifluoroethanol (TFE)/water and 15 mM dodecylphosphatidylcholine (DPC).

10.7717/peerj.5369/supp-2 Supplemental Information 2 Fig. S2. Secondary structures and interactions of cecropin DH with LPS by Fourier-transform infrared (FTIR) spectroscopy. (A) Amide I region of LPS. (B) Antisymmetric phosphate region of LPS. For amide I region measurements (1,750–1,550 cm^−1^), 2 mg/mL LPS in D_2_O alone or with peptide (0.5 mM) were lyophilized and spectra were collected. For the negatively charged phosphate region measurements (1,250–1,150 cm^−1^), similar procedures were performed as for those in amide I region, except H_2_O was selected as the solvent instead of D_2_O.

10.7717/peerj.5369/supp-3 Supplemental Information 3 Raw data.

## Additional Information and Declarations

### Data Availability

The following information was supplied regarding data availability: The raw data are provided in the Supplemental File.
