# Supplementary figures and images for: A novel cecropin B-derived peptide with antibacterial and potential anti-inflammatory properties
Source: PeerJ. 2018 Jul 25;6:e5369. doi: 10.7717/peerj.5369 (PMC6064198; doi:10.7717/peerj.5369)

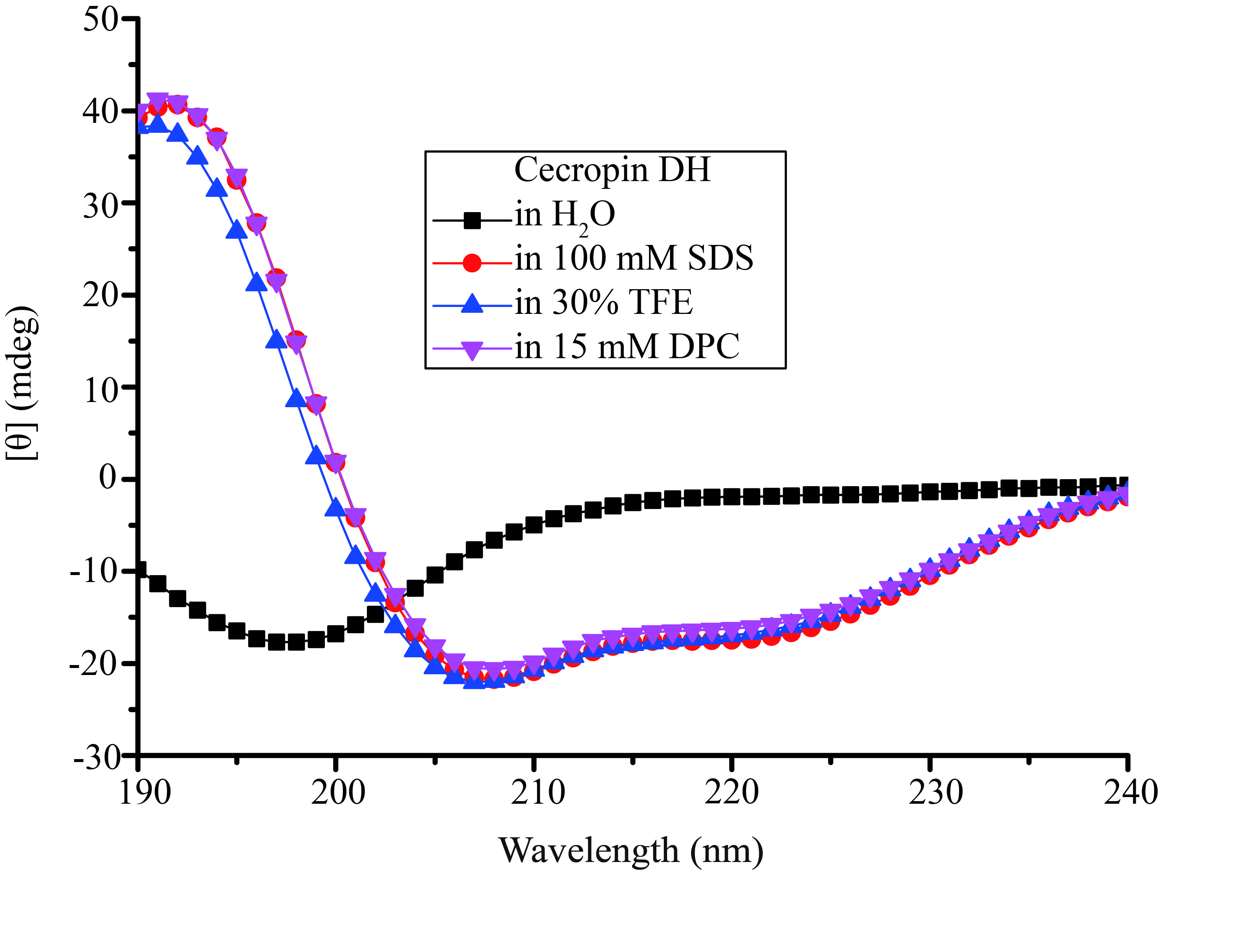

Supplement: Supplemental Information 1 — CD spectra of cecropin DH (0.2 mg/mL) in H2O, 100 mM sodium dodecyl sulphate (SDS), 30% trifluoroethanol (TFE)/water and 15 mM dodecylphosphatidylcholine (DPC). [file peerj-06-5369-s001.jpg]

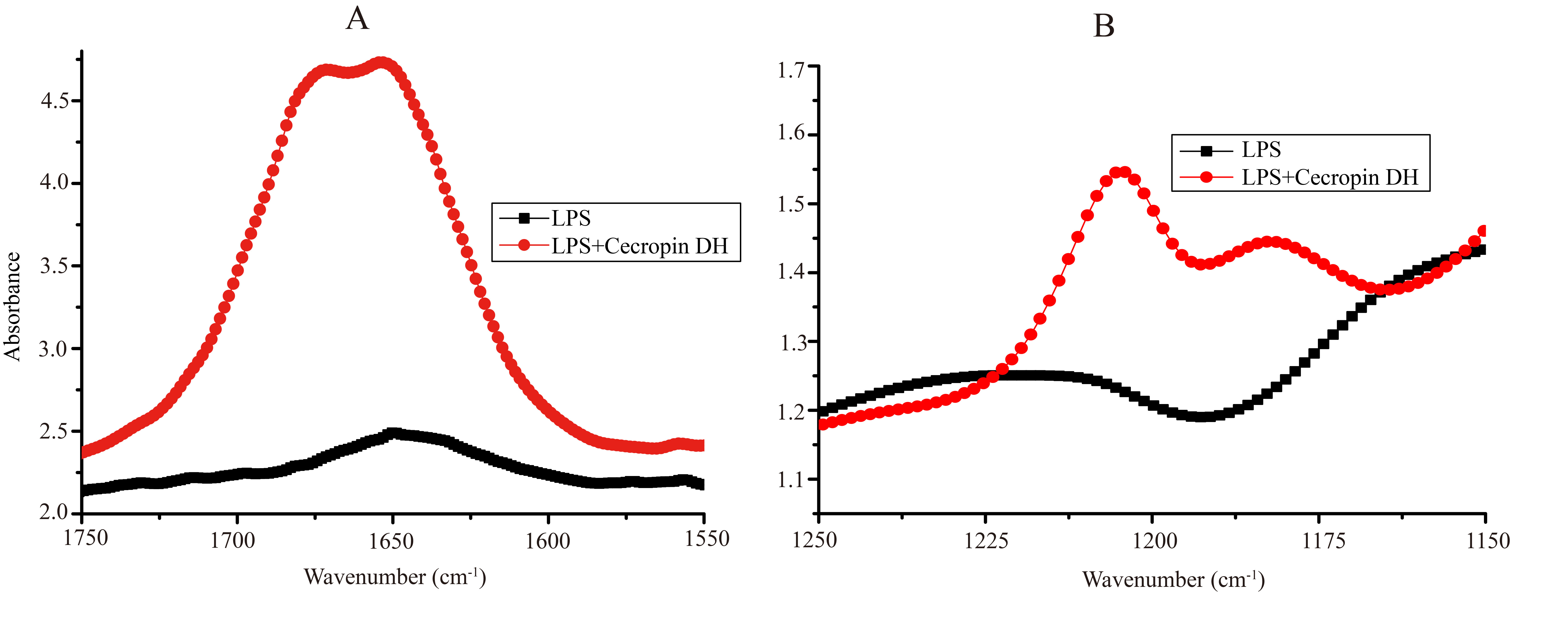

Supplement: Supplemental Information 2 — (A) Amide I region of LPS. (B) Antisymmetric phosphate region of LPS. For amide I region measurements (1,750–1,550 cm−1), 2 mg/mL LPS in D2O alone or with peptide (0.5 mM) were lyophilized and spectra were collected. For the negatively charged phosphate region measurements (1,250–1,150 cm−1), similar procedures were performed as for those in amide I region, except H2O was selected as the solvent instead of D2O. [file peerj-06-5369-s002.jpg]
